# More on mobility and sedentism: Changes in adaptation from Upper Paleolithic to Incipient Jomon, Tanegashima Island, southern Japan

**DOI:** 10.1371/journal.pone.0314311

**Published:** 2025-01-27

**Authors:** Kazuki Morisaki, Fumie Iizuka, Masami Izuho, Mark Aldenderfer

**Affiliations:** 1 Department of Archaeology, Graduate School of Humanities and Sociology, The University of Tokyo, Tokyo, Japan; 2 Department of Anthropology, University of Wisconsin−Madison, Madison, Wisconsin, United States of America; 3 Department of History and Archaeology, Faculty of Social Sciences and Humanities, Tokyo Metropolitan University, Tokyo, Japan; 4 Department of Anthropology and Heritage Studies, University of California, Merced, California, United States of America; Sapienza University of Rome: Universita degli Studi di Roma La Sapienza, ITALY

## Abstract

Sedentism is an adaptive alternative in human societies which is often associated with the emergence of complex societies in the Holocene. To elucidate the factors and processes of the emergence of sedentary societies, continuous accumulation of case studies based on robust evidence from across the world is required. Given abundant archaeological and geological evidence from the late Pleistocene to early Holocene, Tanegashima Island, situated in the southern Japanese Archipelago of the northwestern Pacific Rim, has significant potential to unravel factors and processes of sedentism. Our study evaluates long-term change in hunter-gatherer mobility on Tanegashima Island from the Upper Paleolithic to Incipient Jomon (ca.36,000–12,800 cal BP). Based on Bayesian age modelling, we performed diachronic analyses on lithic toolkit structure, lithic reduction technology, lithic raw material composition, and occupation intensity. The results illustrate that settlement-subsistence strategies on Tanegashima primarily correspond to the change in environmental conditions, mainly food resources, and foragers increased their degrees of sedentism when abundant forest existed. More important is that highly stable sedentism, which is not observed until the Incipient Jomon, depends not only on such a productive environment, but also on the increase in population size. High occupation intensity during the Incipient Jomon on the island is likely attributed to an influx of people from Kyushu proper. Although the relationship between cause and effect of these factors is still to be clarified in future work, our study provides insights on the fundamental causes of sedentism in the temperate forest of the late Pleistocene.

## Introduction

Sedentism is a lifeway with limited or no residential mobility. It is not a threshold phenomenon of social evolution, but an adaptive alternative for human societies [1]. Ethnographic data have furnished pivotal information regarding various patterns, the inherent nature, and causative factors regarding the diversity of settlement mobility [1, 2–7]. Sedentism is often correlated with the latitudinal gradient of environmental properties (e.g., effective temperature) [3, 4, 8–12], and the correlations are understood as adaptive responses to the risks associated with resource distribution and the predictability and the length of the growing season [13].

Archaeologically, the number of sedentary societies often associated with the adoption of pottery and agriculture dramatically increased after the onset of the Holocene [1, 14–19]. However, residentially mobile societies continued to exist, primarily in the Arctic and tropics, well into the Holocene [3, 5, 6, 11, 20–22]. Conversely, artifact assemblages of some Paleolithic societies show signatures of low residential mobility [23, 24]. This suggests that the onset of sedentism cannot be explained simply by environmental proxies such as effective temperatures or latitudes. To better understand the factors leading to sedentism, as well as the correlation between settlement mobility and environment, in-depth data on paleoecology, landscapes, and behaviors in each local context need to be presented [25, 26].

**Fig 1:**
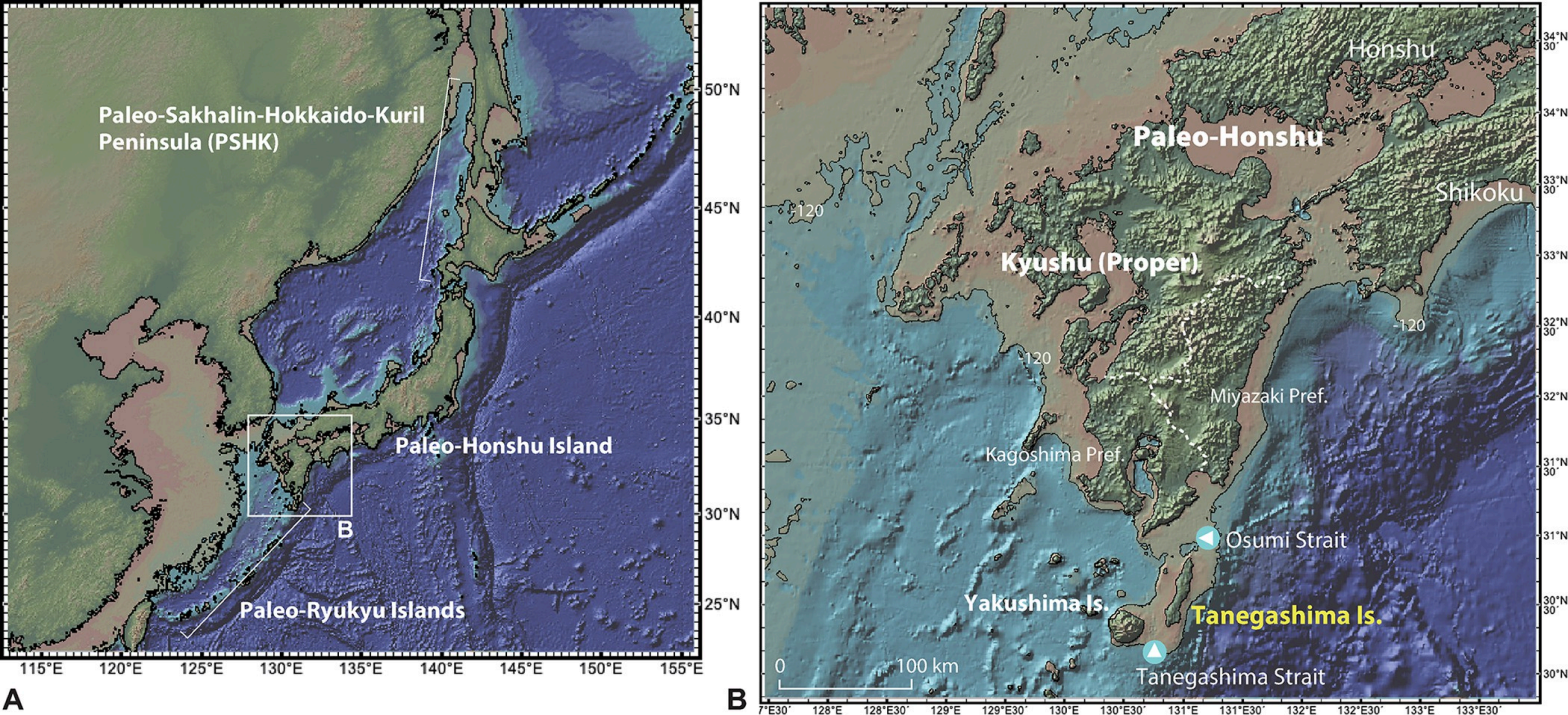
Topographic map of the Japanese Archipelago (A) and Kyushu region (B) during the glacial period. Areas colored in brown indicate bathymetry at ~120 m. The figure was constructed using GeoMapApp (www.geomapapp.org)/CC BY [53].

Thus far, archaeological studies from various contexts around the world have added region-specific conditions that may induce sedentary life [4, 7, 27–34]. To further this understanding, we have developed detailed case studies that examine the relationship between human behaviors, climate, and associated ecosystem changes in several regions on the present Japanese Archipelago of the northwestern Pacific rim, and have estimated the diversity of forager mobility, settlement structure, and sedentism (S1 in S1 File). These investigations encompass periods ranging from the early Upper Paleolithic (EUP) to Incipient Jomon (ICP-J), approximately from 39,000 to 11,500 cal BP [35–52]. Due to the lowered sea level during terminal Pleistocene, the Japanese Archipelago is divided into three major areas: Paleo-Sakhalin-Hokkaido-Kuril peninsula, Paleo-Honshu Island, and Paleo-Ryukyu Islands (Fig 1). Given that several tephra layers form the late Pleistocene geology, which facilitate the evaluation of geochronology related to human occupations, particular emphasis has been placed on southern Kyushu, stretching from the southern edge of Paleo-Honshu and northern part of Paleo-Ryukyu Island. An early onset of increased degrees of sedentism is inferred in southern Kyushu [35–37].

This paper focuses on Tanegashima Island of southern Kyushu. Tanegashima, which today is an offshore island to the south of Kyushu Proper (Kagoshima prefecture) (Fig 1), was on the southwestern periphery of Paleo-Honshu when sea level was lower during the Last Glacial Maximum (LGM). Tanegashima is inferred to lie within the ecotone, bridging the northern edge of the subtropics zone and the southern edge of the warm temperate zone, likely with warm conditions even during the LGM [37, 38, 54]. Tanegashima is known for the construction of deep pits, presumably used for hunting traps, which is unusual for the EUP [55]. It is also one of the earliest regions in the Japanese Archipelago to show significant pottery production by the ICP-J, and the only such place with a secure Pleistocene geochronology [56]. Given this unique situational and archaeological context, the study of mobility strategies and the diachronic changes on Tanegashima provides a valuable test case for understanding the nature and flexibility of local adaptation in the archipelago, as well as for comparisons across the globe, including the beginning of sedentary life and the emergence of pottery production.

## Contexts

### Geography

The geology, geography, and paleoenvironment of the ICP-J in southern Kyushu are detailed in our previous studies [37, 38]. Here we complement them with additional data, especially on the Upper Paleolithic to Incipient Jomon on Tanegashima (ca. 36,000–12,800 cal BP).

**Fig 2:**
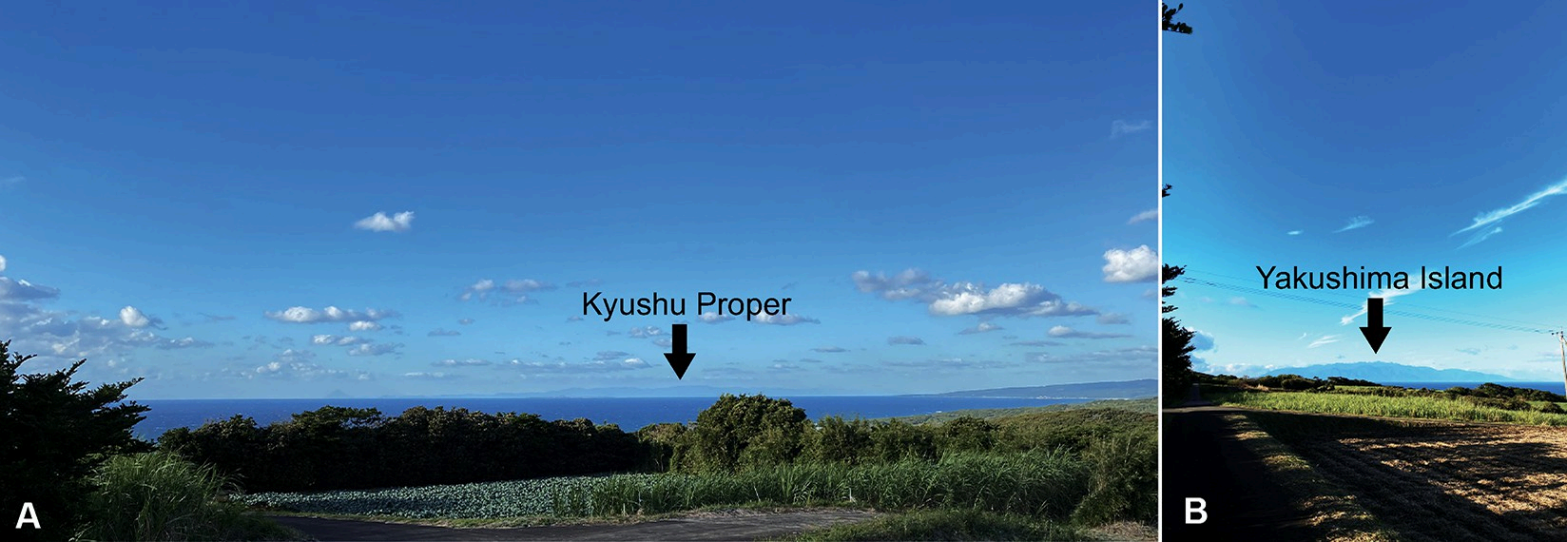
Views of Kyushu Proper (A) and Yakushima Island (B) from Tanegashima Island. Photo by K. Morisaki.

Present-day Kyushu Island was separated from Honshu and Shikoku after the onset of the Holocene [57] (Fig 1). Southern Kyushu comprises the present-day Kagoshima and Miyazaki Prefectures, as well as a chain of smaller islands extending south, including the Osumi Islands, Tokara Islands, and Amami Islands. Tanegashima Island is a major island of the Osumi Islands, located about 33 km south of the Osumi Peninsula with a present-day depth of ca.100 m of the Osumi Strait, and about 17 km northeast of Yakushima Island with a present-day depth of ca. 80 m in the Tanegashima Strait (Fig 2). Tanegashima measures about 57 km northeast to southwest and between 5 and 12 km from east to west, and the area is 447 km^2^. It is composed of hills and plateaus rising up to 282 m.a.s.l. In contrast, Yakushima has a maximum elevation of 1,936 m.a.s.l. [37, 38].

Sea level changes that occurred during the early Upper Paleolithic to Incipient Jomon periods altered the geographic landscape of southern Kyushu. Recent global studies have reconstructed that the global mean sea level of 40–30 kyr ago fluctuated around 70–80 m below the present level, followed by a sudden regression to 120–130 m below the current level between 30–19 kyr ago, during the global LGM [58]. Recent studies supplemented by new data from the Great Barrier Reef revealed that sea level drop shows two steps at 31–29 kyr ago and 22–21 kyr ago, and that it culminated 20.5 kyr ago during the LGM in a global mean sea level low of about −125 to −130 m [59, 60]. Rapid and unidirectional transgression to the present level occurred after that and became more evident after 17 kyr ago. These data suggest that Kyushu proper, Tanegashima, and Yakushima Islands were connected at least during the LGM whereas Tanegashima was disconnected from Kyushu proper before 30 kyr ago. The connection between Tanegashima and Yakushima Islands still requires more in-depth data. These studies are based on multiple dating techniques, including U-series and ^14^C, and are roughly comparable with archaeological radiocarbon chronology.

A study on local boring cores in the north of Kagoshima Bay found that sea level was 85–90 m below the current level around 14,500 cal BP [61]. This suggests that the Osumi Strait may have been submerged by 14,500 cal BP, after 19,000 cal BP, and the Tanegashima Strait between 14,500 and 13,000 cal BP. This assumption needs to be tested by future submarine coring data from the Osumi Strait.

### Geology

**Fig 3:**
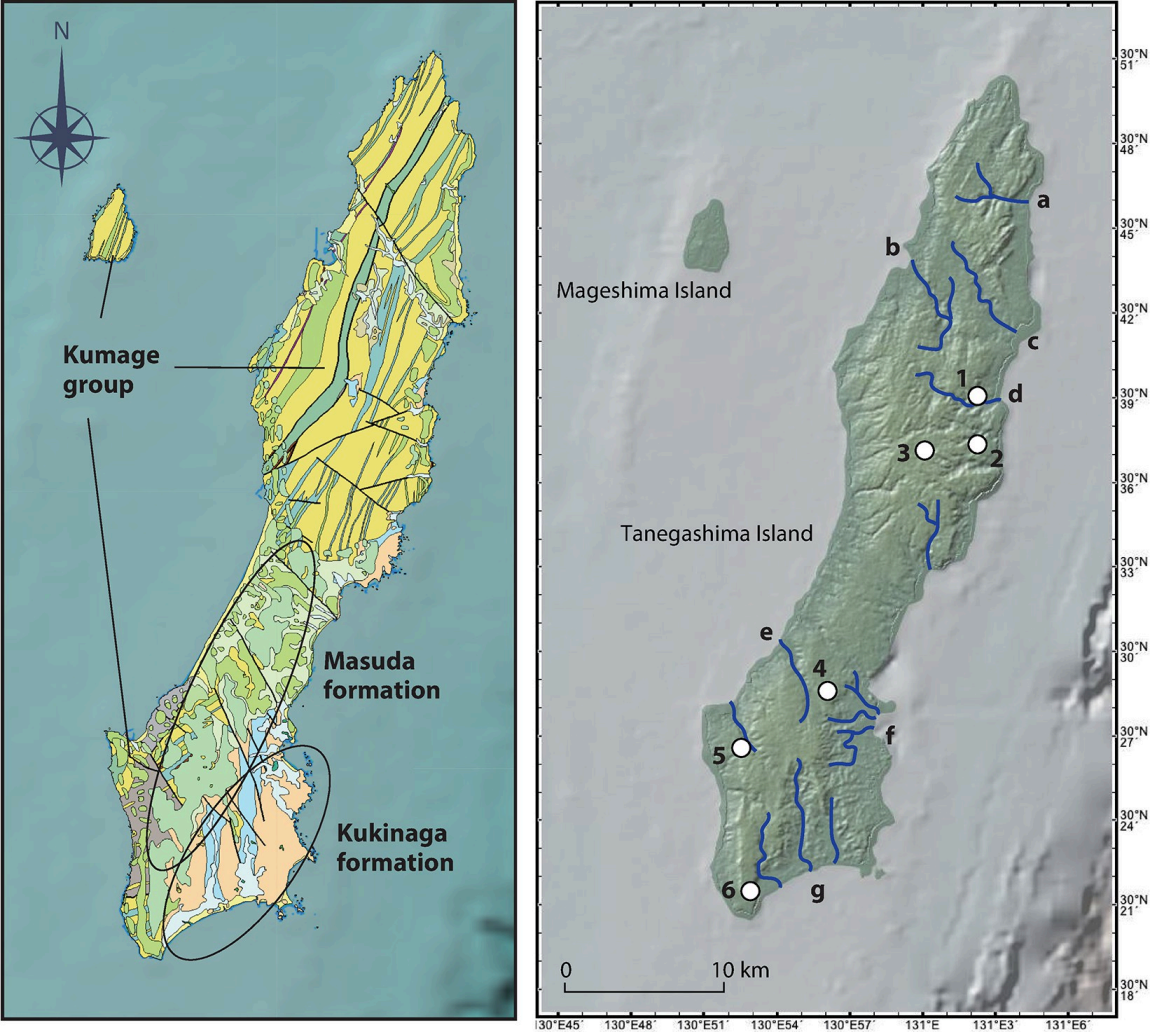
Surface geology and river maps of southern Kyushu with archaeological sites mentioned in the text. The geological map (left) is based on the Seamless Digital Geological Map of Japan V2, provided by the Research Institute of Geology and Geoinformation, AIST (https://gbank.gsj.jp/seamless/)) [66]. In the map on the right, sites mentioned in this text and rivers with drainage basin exceeding 10 km^2^ are labelled; 1: Onigano site, 2: Okunonita site, 3: Sankakuyama I site, 4: Tachikiri site and Naganosaki site, 5: Yokomine C site, 6: Zenigame site. a: Saikyo riv. b: Koume riv. c: Minato riv. d: Kawawaki riv. e: Kubama riv. f: Oura riv. g: Kori riv.

As detailed in the previous study [38], the basal geology of Tanegashima Island comprises an accretionary complex with Eocene and Oligocene mudstones and sandstones, named the Kumage group within the upper Shimanto supergroup (Fig 3) (S2 in S1 File) [62–64]. Tanegashima Island lacks plutonic, obsidian, or andesite outcrops, which simplifies the identification of both locally produced and exotic artifacts found on the island. Among the existing bedrock, small pebbles (φ<10 cm) of hard shale, mudstone, and hornfels are of knappable quality [65].

Major late Pleistocene tephra mentioned in this paper include tephra layers of Tane-III, Tane-IV, Aira Tn Tephra (AT), and Sz-S from the bottom to the top. Tane III and Tane IV are composed of fine ashes that blanket the entirety of Tanegashima Island (S2 in S1 File). Their ages are estimated to be 45 ka for Tane III, and 35 ka for Tane IV [67–70]. The AT, which erupted from the Aira Caldera located in northern Kagoshima Bay (Fig 1), has been precisely dated to 30,000 cal BP by several dating methods [69–71]. AT is widely distributed throughout the Japanese Archipelago, the Sea of Japan, the southern Korean Peninsula, the East China Sea, and the Pacific Ocean Basin [68]. Sz-S, originating from Sakurajima Volcano on the southern somma of Aira Caldera, is dated to 12,800 cal BP [69, 70].

### Paleoenvironment

Presently, the southernmost region of Kyushu proper and northeastern Tanegashima experience an annual average precipitation ranging from about 2000 to 2800 mm. However, in areas such as the southern tip of the Osumi Peninsula, southern Tanegashima, and the lowland Yakushima, the precipitation is greater, ranging from about 3000 to 4000 mm annually. The highest precipitation, exceeding 8300 mm, is observed in the higher elevations of Yakushima [37, 38, 72]. It is inferred that the southern tip of Kyushu Island, Tanegashima, and Yakushima were warmer than the northern part of southern Kyushu. Regardless of this high precipitation, obtaining water on Tanegashima is not easy due to the island’s small size and narrowness. Only seven rivers have relatively wide drainage basins exceeding 10 km^2^, and they are distributed separately in the northeastern and southwestern parts of the island. People living in the central part of the island likely relied on occasional groundwater springs until modern-day waterworks were established [73] (Fig 2).

Mainly due to the Kuroshio Ocean Current, southern Kyushu experienced a warm climate even during the LGM. This region was covered by warm-temperate species, including temperate coniferous forests and temperate deciduous broad-leaved mixed forests. The lowlands of the southern tip of Kyushu proper, then connected with Tanegashima and Yakushima, also had warm-temperate evergreen forests and broad-leaved evergreen forests [35, 54]. These warm and wet environments likely enhanced biodiversity and primary production [38]. Based on its present distribution and DNA haplogroups [54], it is inferred that southern Kyushu served as a refugium for broad-leaved evergreen forests including *Castanopsis sieboldii* even during the LGM [74].

However, at present, no signatures of evergreen flora are found in archaeological site-based pollen analysis from the LGM [75]. They are found only in contexts before and after the LGM. This implies that warm indicators were likely weakened during the LGM, even in these areas.

## Materials and methods

### Sites and materials

This study focuses on late Pleistocene sites on Tanegashima island and references interrelated phenomena in Kyushu proper to obtain behavioral inferences. We present sites and materials from both islands and offer concrete data sets from Tanegashima.

A total of 2,855 Paleolithic and ICP-J sites including surface collections have been found in Kyushu, the southwestern edge of Paleo-Honshu [76]. Of these, 898 sites are located in southern Kyushu. The Upper Paleolithic chronology in Kyushu proper is classified into three phases: early, middle, and late. The EUP begins around 39–37,000 cal BP below AT and is characterized by trapezoids on flakes and edge-ground axes/adzes. The adoption of blade-based backed points occurs later, around 34,000 cal BP. The Middle Upper Paleolithic (MUP: 30,000–19,000 cal BP) begins above the AT. It coincides with the LGM and features varied backed points and pointed tools on flakes along with thick blades made of high-quality lithic raw materials such as siliceous shales. Microblade technology emerges in the Late Upper Paleolithic (LUP: 19,000–16,000 cal BP). The oldest pottery adoption in this region could date as early as 16,000 cal BP.

On Tanegashima Island, a total of 13 late Pleistocene archaeological sites have been found through general surveys, testing, and rescue excavations (Fig 3). Some of the sites are multi-component sites. We use the term “component” to refer to an archaeological unit at a single site in a region.

**Fig 4:**
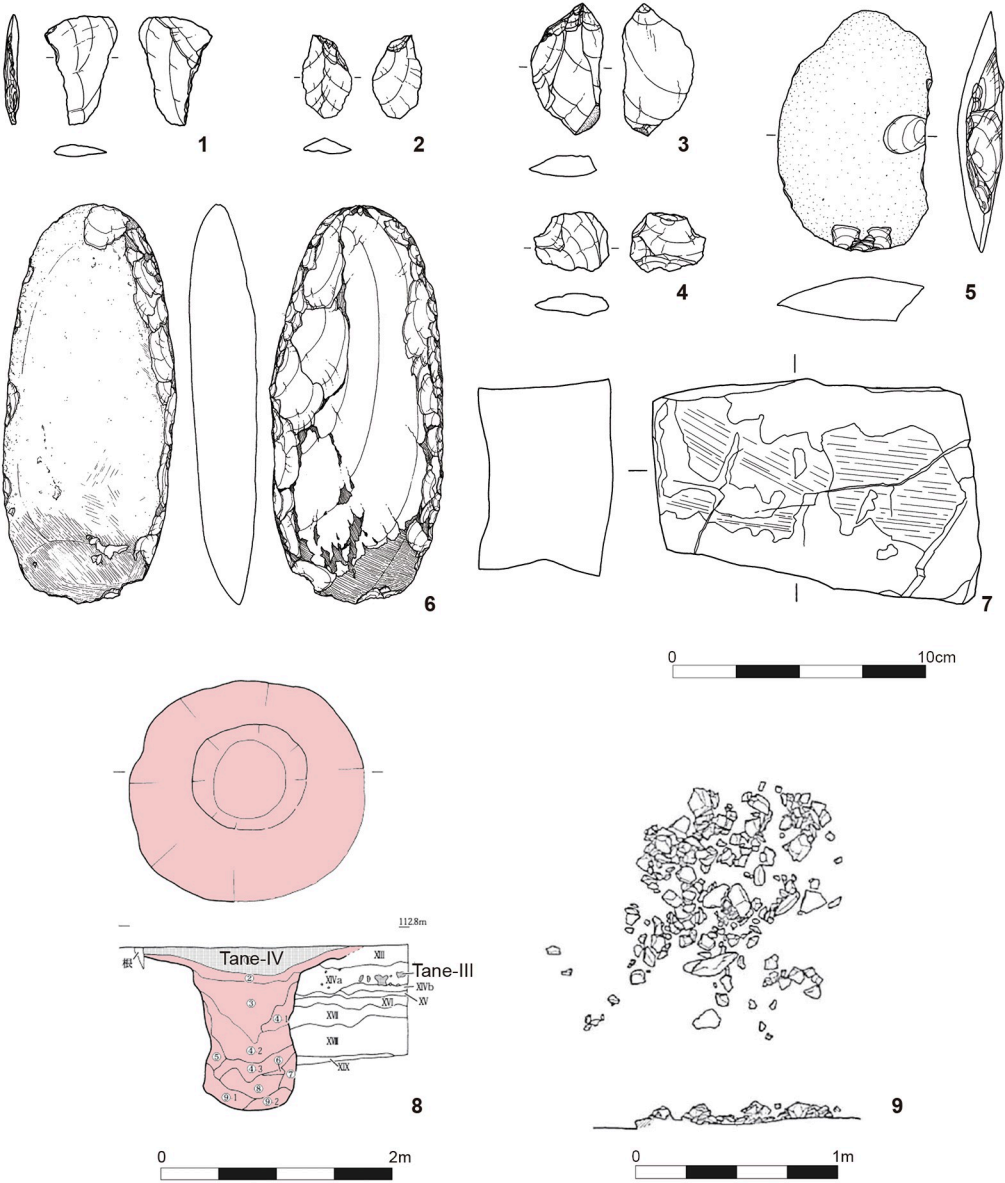
Artifacts and features of the early Upper Paleolithic on Tanegashima. 1–8: Tachikiri site: CL1, 9: Yokomine C site; CL1, 1: trapezoid, 2–4: flakes, 5: chopper, 6: edge-ground axe, 7: wet stone, 8: pit-trap, 9: pebble aggregate.

The oldest evidence for the appearance of modern human on Tanegashima is found below the Tane-IV (35 ka) tephra at the Tachikiri site and Yokomine C site. This includes small numbers of retouched flake tools, pebble tools such as chopper-chopping tools, grinding stones, and stone plates, all made of sandstones [37, 77, 78]. Several pit-traps were found at the Tachikiri site (Fig 4). Starch granules observed on the working surfaces of grinding stones indicate that they were used for plant processing [79]. The second oldest components, found between the Tane-IV and AT tephras at these two sites, comprise retouched flake tools and various pebble tools but lack pit-traps. The summary report of the excavation concluded that these components date to around 35,000 cal BP [80]. These components predate the LGM and are assigned to the EUP. Unlike Kyushu proper, no blade technology has been identified in the EUP on Tanegashima.

**Fig 5:**
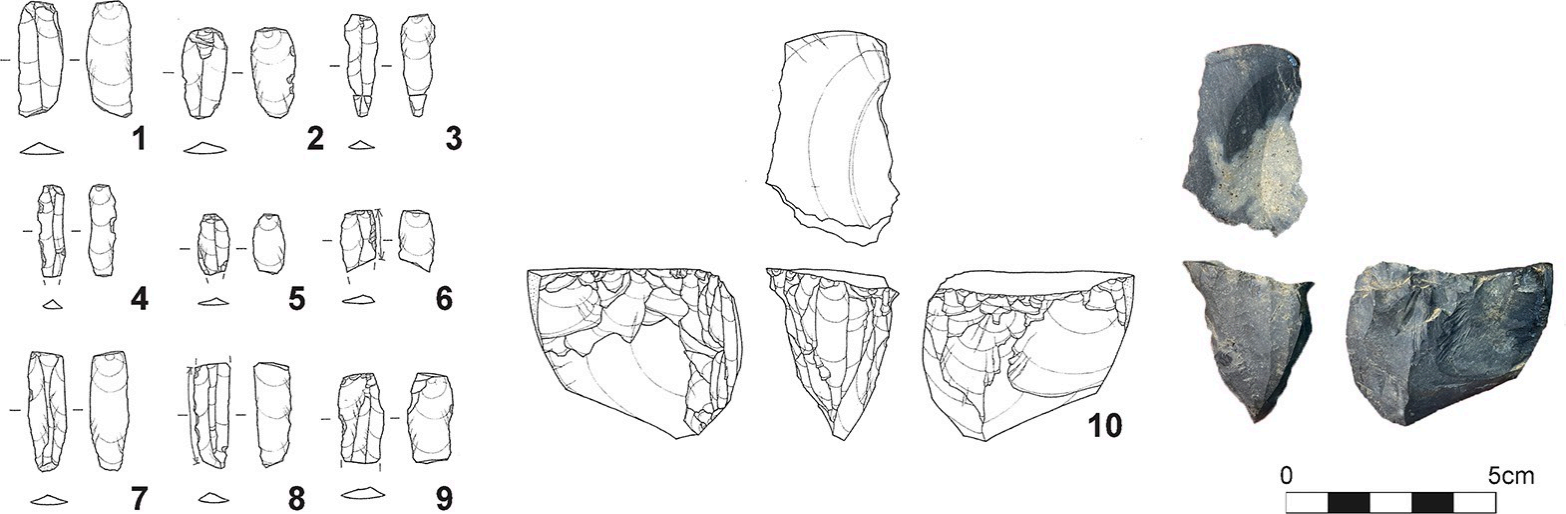
Artifacts of the Late Upper Paleolithic on Tanegashima. 1–10: Naganosaki site; 1–9: microblades, 10: microblade core.

Currently, no archaeological sites from the LGM have been identified on Tanegashima. After the LGM, terminal LUP foragers equipped with microblade toolkits occupied Tanegashima before the Incipient Jomon (Fig 5). Four LUP components found between the AT and Sz-S tephras have not been radiocarbon-dated. However, their age can be tentatively inferred from radiocarbon dates of microblade assemblages at the Kiriki site in southern Kyushu [81], ranging from 17,800 to 16,100 cal BP based on IntCal20 [82, 83].

**Fig 6:**
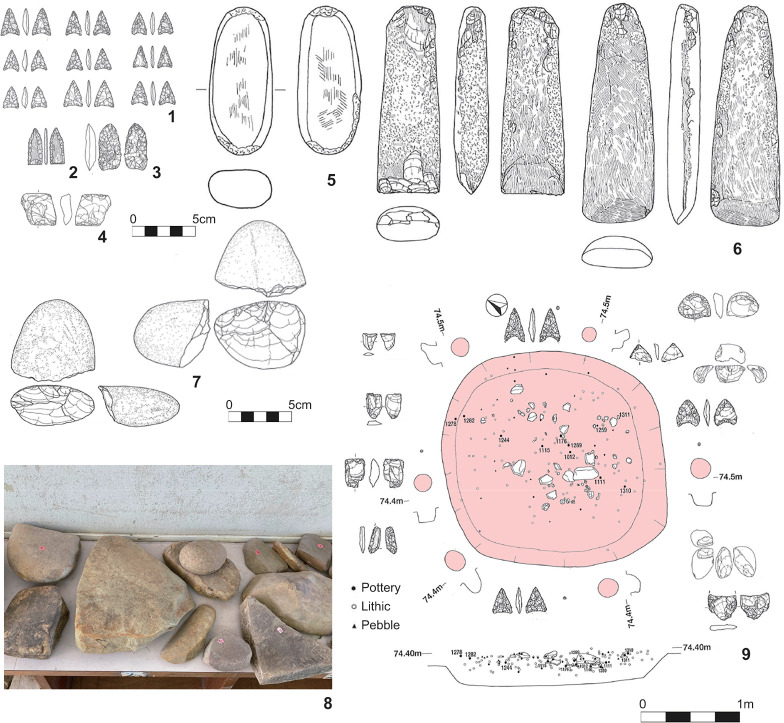
Artifacts and features of the Incipient Jomon on Tanegashima. 1–9: Onigano site; 1: chipped arrowhead, 2: ground arrowhead, 3: bifacial point, 4: wedge, 5: hammer stone/grinding stone, 6: ground axes, 7: choppers, 8: various pebble tools—stone plates, grinding stones, anvil, hammer stones, 9: pit-house.

The ICP-J component is found immediately below the Sz-S tephra, at nearly the same stratigraphic level as in southern Kyushu. Currently, 11 ICP-J sites, including surface collections, have been identified on Tanegashima [84]. This period saw a dramatic increase in pit-houses, burnt pebble aggregates likely used as cooking facilities, numerous pebble tools, and potsherds (Fig 6). The calibrated dates of the ICP-J range between ca.14,000/13,500–12,800 cal BP, aligning with the Bølling/Allerød oscillation (ca. 14,700–12,900 cal BP) [37–39, 43, 44, 56]. Petrographic analysis and electron microprobe and neutron activation studies of pottery from ICP-J sites on Tanegashima suggest that most pottery was produced on the island, with some pieces originating from Yakushima Island and/or southern Kyushu Proper [37, 38]. Pottery producers are hypothesized to have been sedentary hunter-gatherers living in a warm and wet environment, engaging in costly communication and exchange to mitigate subsistence and social reproduction risks [37, 38].

We selected 15 components with sufficient data for analyses from EUP to ICP-J sites on Tanegashima (Table 1). Of these sites, four localities (loc.) of the Tachikiri site─loc. Tachikiri, loc. Otsubobata, loc. Kozono, and loc. Imahira-Shimizu─and the Yokomine C site are multi-component and include several components from below Tane-IV to above AT. For the following analyses, data on the composition of assemblages, toolkits, reduction methods, raw materials, and archaeological features were collected from excavation reports (Table 1), with some revisions based on our personal observations. All reported radiocarbon dates pertaining to these components are listed in Table 2.

**Table 1:** General information of the sites analyzed in this paper.

Phase	Site		Excavation area (m^2^)	Geologic layer	Stratigraphic position	Lithics (pieces)	Potsherds (pieces)	Reference
<Sz-S (12.8 ka ago)>								
ICP-J		Onigano	4400	VI	below Sz-S	3822*	14352	[85]
		Okunonita	1000	V	below K-Ah, above AT	317*	~1500	[86]
		Sankakuyama I	58620	V	below Sz-S, above AT	710	~4000	[87]
LUP		Zenigame	3000	IV	below K-Ah	c.100~200	n/a	[88]
		Tachikiri; loc.Tachikiri/CL3	1050	V	below K-Ah, above AT	c.200~300	n/a	[89]
		Tachikiri; loc.Otsubobata/CL3	1400	V	below K-Ah, above AT	13	n/a	[90]
		Naganosaki	67.5	V	below K-Ah	553	n/a	[91]
<AT (30 ka ago)>								
EUP-l		Tachikiri; loc.Otsubobata/CL2	1400	XI	below AT, above Tane-IV	16	n/a	[90]
		Tachikiri; loc.Kozono/CL2	1400	XI	below AT, above Tane-IV	8	n/a	[90]
		Tachikiri; loc.Imahira &Shimizu/CL2	c.540	XI	below AT, above Tane-IV	16	n/a	[80]
		Yokomine C/CL2	2117	XI	below AT, above Tane-IV	13	n/a	[92, 93]
<Tane-IV (35 ka ago)>								
EUP-e		Tachikiri; loc.Tachikiri	c.2000	XIII	below Tane-IV, above Tane-III	<400	n/a	[80, 89, 94, 95]
		Tachikiri; loc.Otsubobata/CL1	1400	XIII	below Tane-IV, above Tane-III	0	n/a	[90]
		Tachikiri; loc.Imahira &Shimizu/CL1	c.540	XII, XIII	below Tane-IV, above Tane-III	7	n/a	[80, 96]
		Yokomine C/CL1	2117	XIII	below Tane-IV, above Tane-III	37	n/a	[92, 93]
<Tane-III (45 ka ago)>								

*The total number of lithic specimens was revised from the report based on the authors’ recount.

**Table 2:** Radiocarbon dates obtained from sites mentioned in this paper.

	Site	Layer	Sample	Method	Lab-code	^14^C age (BP)		δ^13^C		evaluation	Reference
1	Onigano	VI(PH1)	Charcoal	AMS	IAAA-11504	10470	60	-30.3	0.7	reject (outlier)	[85]
2	Onigano	VI	CRP*	AMS	IAAA-11505	9220	50	-21.9	0.7	reject (outlier)	[85]
3	Onigano	VI	CRP	AMS	Beta-177290	12180	40	-22.4	-	accept	[85]
4	Onigano	VI	CRP	AMS	Beta-177289	11880	60	-23.2	-	accept	[85, 97]
5	Onigano	VI	CRP	AMS	MTC-09138	11990	60	-27.0	1.6	accept	[85]
6	Onigano	VI	CRP	AMS	MTC-09139	12130	60	-26.6	1.7	accept	[85]
7	Okunonita	unknown	CRP	AMS	MTC-09141	11740	60	-29.4	1.5	reject (context unknown)	[86]
8	Sankakuyama I	V	CRP	AMS	IAAA-10309	11370	70	-30.9	1.5	accept	[87]
9	Sankakuyama I	V(PH1)	CRP	AMS	IAAA-10310	11530	60	-24.5	1.3	accept	[87]
10	Sankakuyama I	V(PH1)	CRP	AMS	IAAA-10311	11950	70	-29.1	1.5	accept	[87]
11	Sankakuyama I	V(PH1)	Charcoal	AMS	Beta-175701	11640	50	-26.6	-	accept	[87]
12	Sankakuyama I	V(PH1)	CRP	AMS	Beta-175702	11940	70	-22.3	-	accept	[87]
13	Sankakuyama I	V	CRP	AMS	IAAA-31693	11472	67	-25.3	0.9	accept	[87]
14	Sankakuyama I	V	CRP	AMS	IAAA-31694	12049	72	-21.6	1.0	accept	[87]
15	Sankakuyama I	V	CRP	AMS	IAAA-31695	12094	67	-21.8	0.7	accept	[87]
16	Sankakuyama I	V	CRP	AMS	IAAA-31696	11664	66	-22.7	1.1	accept	[87]
17	Sankakuyama I	V	CRP	AMS	IAAA-31697	11052	64	-27.5	0.9	accept	[87]
18	Sankakuyama I	V	CRP	AMS	MTC-05834	12080	70	-24.8	-	accept	[87]
19	Sankakuyama I	V(PA)	Charcoal	AMS	Beta-105040	11100	80	-24.0	-	accept	[98]
20	Sankakuyama I	V(PA)	Charcoal	AMS	Beta-105041	11040	80	-24.6	-	accept	[98]
21	Sankakuyama I	V(PA)	Charcoal	AMS	Beta-105042	11140	80	-23.4	-	accept	[98]
22	Sankakuyama I	V(PA)	Charcoal	AMS	Beta-105043	12120	80	-20.3	-	accept	[98]
23	Sankakuyama I	V	CRP	AMS	Beta-125372	12260	50	-23.2	-	accept	[98]
24	Sankakuyama I	V	CRP	AMS	Beta-125373	11800	50	-26.4	-	accept	[98]
25	Sankakuyama I	V(PA)	Charcoal	AMS	Beta-125374	10990	120	-25.4	-	accept	[98]
26	Sankakuyama I	V	Charcoal	AMS	Beta-88847	11880	60	-27.1	-	accept	[87]
27	Sankakuyama I	V	Charcoal	Conventional	Gak-19077	10470	170	-	-	reject (conventional)	[87]
28	Sankakuyama I	V	CRP	AMS	Beta-153028	8710	90	-26.2	-	reject (outlier)	[87]
29	Sankakuyama I	unknown	Charcoal	AMS	Beta-99999	10000	100	-25.0	-	reject (context unknown)	[87]
30	Sankakuyama I	V	CRP	AMS	PLD-6470	11790	45	-23.4	0.3	accept	[87]
31	Sankakuyama I	V	CRP	AMS	PLD-6471	11795	50	-45.5	0.3	accept	[87]
32	T. loc. Tachikiri; CL1	XId(CC)	Charcoal	AMS	IAAA-110764	30254	112	-26.1	0.5	accept	[80]
33	T. loc. Tachikiri; CL1	Xid(BS)	Charcoal	AMS	IAAA-110765	30045	115	-25.1	0.6	accept	[80]
34	T. loc. Tachikiri; CL1	XIII(PAt4)	Charcoal	AMS	IAAA-110766	30366	117	-24.5	0.6	accept	[80]
35	T. loc. Tachikiri; CL1	XIIIa	Charcoal	AMS	IAAA-110767	30929	116	-24.0	0.5	accept	[80]
36	T. loc. Tachikiri; CL1	XIII(BS)	Charcoal	AMS	Beta—114267	30480	210	-26.3	-	accept	[94]
37	T. loc. Tachikiri; CL1	XIII	Charcoal	AMS	Beta—169709	30390	600	-24.3	-	rejected (large error)	[95]
38	T. loc. Tachikiri; CL1	XIII	Charcoal	AMS	Beta—169710	28420	500	-26.4	-	rejected (large error)	[96]
39	T. loc. Otsubobata;CL2	Xupper(CC)	Charcoal	AMS	IAAA-71653	30321	165	-25.3	0.8	accept	[90]
40	T. loc. Otsubobata;CL2	Xupper(BS)	Charcoal	AMS	IAAA-71654	30109	185	-20.4	0.7	accept	[90]
41	T. loc. Otsubobata;CL2	Xupper(CC)	Charcoal	AMS	IAAA-71655	29305	166	-25.7	0.8	accept	[90]
42	T. loc. Kozono;Cl.2	IX(PA)	Charcoal	AMS	IAAA-71656	28216	142	-24.1	0.8	accept	[90]
43	T. loc. Kozono;Cl.2	IX(PA)	Charcoal	AMS	IAAA-71657	27957	144	-24.6	0.8	accept	[90]
44	T. loc. Kozono;Cl.2	IX(PA)	Charcoal	AMS	IAAA-71658	29444	141	-22.4	0.9	accept	[90]
45	T. loc. Kozono;Cl.2	IX(PA)	Charcoal	AMS	IAAA-71659	29745	158	-22.7	0.7	accept	[90]
46	T. loc. Kozono;Cl.2	IX(PA)	Charcoal	AMS	IAAA-71660	29040	163	-21.7	0.6	accept	[90]
47	T. loc. Kozono;Cl.2	IX(PA)	Charcoal	AMS	IAAA-71661	27509	125	-25.7	0.7	accept	[90]
48	Yokomine C; CL.1	XII(PA)	Charcoal	AMS	Beta-102399	31280	690	-25.8	-	rejected (large error)	[92]
49	Yokomine C; CL.1	XII(PA)	Charcoal	AMS	Beta-102400	29670	540	-24.6	-	rejected (large error)	[92]
50	Yokomine C; CL.1	XII	Charcoal	AMS	Beta-102401	30490	590	-24.3	-	rejected (large error)	[92]
51	Yokomine C; CL.2	XI(PA)	Charcoal	AMS	Beta-102402	29300	520	-25.2	-	rejected (large error)	[92]

“Tachikiri site” is abbreviated as T.

*CRP: charred residue on pottery.

## Methods

### Theoretical perspectives referenced

Anthropological and adaptation models on forager mobility and sedentism inform consideration of external factors affecting decision making [1, 3–5, 9, 11–13, 99–109]. It is well-accepted that risk management related to resource exploitation was the primary driver of foragers’ decision making [9, 13, 17].

We consider two basic models of forager mobility: the forager-collector model and the traveler-processor model. Foragers and collectors have been differentiated by strategy of residential and logistical movement in given environmental contexts [3, 4, 110–115]. Foragers equipped with simple tools frequently move their residential bases to homogeneous resource patches. Collectors procure specific resources from a relatively stable residential base in highly seasonal environments with more complex procurement technologies. Foragers are associated with immediate return systems and collectors with delayed-return systems [116]. However, the traveler and processor model [110, 111] has more predictive power, because it is based on behavioral ecological models which target not specific but instead a wide spectrum of resource contexts typified by a patch choice model or a diet breadth model [110, 111, 117, 118]. In the traveler model, when resources are abundant for a small population, human groups can mostly target highly-ranked resources and spend more time in traveling between those patches. By contrast, high population density makes it difficult to focus solely on just on high-ranked resources and tends to force them to expand the diet breadth to lower-ranked resources. Human groups spend less time traveling between patches, and rather expand search or procurement time, resulting in longer processing time in more reduced residential mobility.

The traveler and processor model also matches other studies on tool procurement contexts that link mobility and tool manufacturing for resource extraction [119–124]. Highly mobile people are equipped with a small number of curated tools such as bifacial tools, or tools featuring standardized components such as blade tools and microblades. Toolkit diversity is limited, and each tool tends to have multifunction [5, 9, 45]. In contrast, tools of sedentary people exhibit a propensity for specialization with designs tailored to specific tasks, thus resulting in more complex toolkits including pottery [9, 13, 121]. Pottery production is often regarded as indicative of sedentism [125].

All these models suggest that data on toolkit diversity, reduction strategy, and lithic raw material procurement strategies provide insights into mobility strategy. Furthermore, data on toolkit diversity is useful also in measuring resource extraction strategies or target resource breadth. Lastly, data on occupational intensity at a site could be an indicator of sedentariness or patch exploitation time.

Other recent models add nuance to these fundamental frameworks. Niche construction models [126] consider settlement system changes [126–129], and it has been posited that energy-optimizing mobility decisions of hunter-gatherers could be influenced not only by natural environmental condition, but also by the artificial accumulation of material resources at places on landscapes due to their prior occupations and activities [126]. It should be noted that the presence of previously occupied sites encourages recurring occupation to reduce costs of construction of several facilities [130].

### Bayesian age modelling

To ensure accurate comparison between forager mobility and paleo-environmental data, we meticulously verify the geochronology of all sites using radiocarbon chronology. After confirming consistency between 51 corrected ^14^C dates and tephrochronology, we discard conventional dates, obvious outliers, dates from unknown geological contexts, and dates with a one-tailed standard error exceeding 400 years from those listed in Table 2 [47, 131]. Calibrated dates and Bayesian age models of each geochronological phase (early EUP: below Tane-IV, late EUP: between Tane-IV and AT, LUP: between AT and ICP-J, and ICP-J: below Sz-S) are then constructed using OxCal 4.4 [82]. For the summary of each phase, we utilize the KDE Model algorithm of OxCal 4.4 to generate a kernel density distribution chart of the dates. The KDE Model command applies the Silverman bandwidth estimate to calculate the kernel density distribution, graphically depicting trends in the distribution of the date series [132].

### Toolkit structure

We categorize lithic tools into fundamental types [37, 77, 78] that belong to several functional tool categories: hunting weapons, processing tools, tree-felling tools, and pebble furniture. We use the functional categories to apprehend the variety of subsistence activities conducted during each phase. Therefore, the classification is based simply on the experimental use-wear analysis and ethnographic reference if such a source exists. Hunting weapons include arrowheads, projectile points, and composite weapons such as microblades. Trapezoids are also assigned to the hunting weapon category based on recent experimental use-wear analyses [133]. Processing tools include denticulates and scrapers. Tree-felling tools encompass axes and adzes along with choppers or chopping tools [134]. Other pebble tools are classified as pebble furniture, a type of site furniture [101], because they are generally large and heavy and assumed to represent non-mobile tools.

Here we adopt two indices: toolkit richness and evenness. Toolkit richness indicates the number of tool types present. When a component possesses chipped arrowheads, end scrapers, drills, and hammerstones, the richness is counted as four. The degree to which each tool type is equally represented is calculated as evenness by using *Simpson’s diversity index* (SDI) [135, 136]. Here, SDI serves as a proxy for the degree of specialization in carrying out specific activities [44, 137]. The SDI formula can be expressed in several ways. The paper adopts the following:

$$SDI=1-\sum_{i=1}^{s}Pi^{2}$$ (1)

Where *S* is the total number of tool types (richness), and *Pi* is the relative abundance of tool type *i*. Measurement of SDI in archaeological contexts is useful for mitigating unusual increases in toolkit richness, and confirming the stability of toolkit diversity. The greater the SDI value, the more evenly individual tool types are represented in a given component, indicating higher specialization of each tool type. An increased level of specialization is interpreted to correlate with elevated degrees of sedentism. Toolkit diversity for each component can be shown as a standardized value, calculated by multiplying richness by evenness (SDI). This paper refers to the value as the ‘RE value (REV)’. The inter-site variability of REV can also indicate the differentiation of subsistence activities.

### Lithic technology and lithic raw material

Five lithic technologies are identified on Tanegashima: flake reduction (e.g., bipolar technique), pebble tool reduction, ground tool reduction, biface reduction, and microblade reduction. To assess the frequency of each reduction strategy, we count the number of each tool type categories and convert these counts into percentages of the entire lithic assemblage. Flake, pebble tool, and ground tool reductions are less suitable for high mobility settlement systems compared to microblade reduction. Pebble tools are made from sandstones collected in the vicinity each site. Microblade and biface reductions are curated and are generally more suitable for mobile lifeways than simple flake and pebble tool reductions [120].

Tanegashima Island has abundant sandstones from the Kumage group, found from the hillsides to the coast, and well-rounded shale, mudstone, and hornfels possibly originating from the conglomerate of the Kukinaga group in the southeast. Most lithic raw materials are well-sorted sandstone, mudstone and hornfels locally procured from Tanegashima. Plutonic rocks, obsidian, and andesite are absent and could not have been locally acquired [37, 38]. We confirm the presence or absence of exotic lithic raw materials to assess the mobility range or exchange.

### Occupational intensity

We examine the total amount of artifacts as an index of occupation intensity within single components of individual sites. To validate this index, we check for potential bias due to the size variability of excavated areas and site discovery methods [138]. We analyze the relationship between excavated area and assemblage size, represented by the number of recovered artifacts.

The degree of occupation intensity is explored from the composition of archaeological features and the labor costs devoted to their construction. Different features, requiring varying labor costs [130], serve as proxies for occupation intensity [44, 46, 139]. To estimate this, we categorize archaeological features into five ranks based on labor cost investment, following the data provided by Morgan et al. [130].

Pit-houses and pit-traps entail high labor cost for digging earth. Morgan et al. [130] experimentally excavated subsurface features of two pit-houses (House-pit 1: 3*3m plan with a depth of 0.22m, House-pit 2: 3.5*3.5 m plan with a depth of 0.31m), reporting energetic expenditure of 2831 kcal and 5105 kcal, respectively. The average size of ICP-J pit-houses excavated on Tanegashima is approximately at least 2/3 volume of House-pit 1, indicating no less than 2000 kcal of energetic expenditure even for just subsurface construction. Furthermore, Morgan et al. [130] reported the construction energy for three storage pits (each with an approximate volume of 0.02 m^3^ for Storage Pit A, 0.04 m^3^ for Storage Pit B, and 0.06 m^3^ for Storage Pit C) as 141, 110, and 163 kcal, respectively. The approximate volume of pit-traps from the Tachikiri site loc. Otsubobata ranges between 0.33 and 0.82 m^3^ (0.56 m^3^ on average) (calculated from 12 metric data of pit-traps provided by the Kagoshima Prefectural Archaeology Center [90], implying ten times the labor cost of the experimental storage pit. The energy expenditure for constructing pit-traps at the Tachikiri site is estimated to be between 1000–2000 kcal. Based on these data, pit-houses require the highest labor cost investment (labor cost score (LCS) 4 per feature), and pit-traps require the second highest (LCS 3 per feature). Pebble aggregates and stone alignments are not reported by Morgan et al. [130] but are inferred to incur smaller labor costs because construction materials can be transported from the vicinity of the site. This paper evaluates the energetic expenditure for constructing pebble-aggregate and stone-alignment as lower than for features involving earth excavation (LCS 2 per feature). Similarly, small pits (0.1–0.2 m in diameter) require minimal energy investment, probably less than 1000 kcal (LCS 1 per feature). Burned soils or charcoal concentrations, which result from simple hearths, are evaluated as requiring little to no investment (LCS 0).

Site furniture and caches are considered as insurance gear [101]. These usually correlate with a highly repetitive settlement system or ‘collecting system’ [3, 101], and with lower residential mobility. However, the direct application of this prediction requires caution because the longevity of the behavior associated with house foundations may extend well beyond the time people abandoned the site [140]. Although this caution is noted, tools recycled or used for multi purposes can indicate intensified or recurring occupation [126]. Thus, we use the percentage of recycled or multi-functional tools, along with the total weight of pebble tools, to supplement the above method for evaluating occupation intensity.

## Results

### Radiocarbon chronology

**Fig 7:**
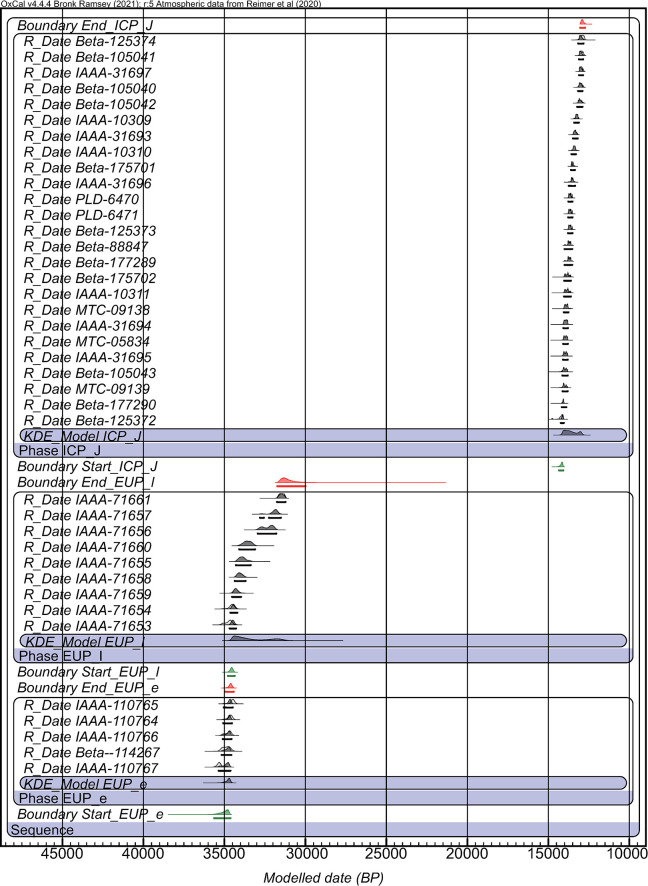
Bayesian age model for EUP to ICP-J components.

Our data ‘hygiene’ process rejected one conventional date, three obvious outliers, two dates from unknown stratigraphic context, and six dates with one-sided standard error exceeding 400 years (see ‘evaluation’ in Table 2). The age model on the cleaned data is provided in Fig 7. Our result clarified that the EUP can be divided into two phases. The early EUP components (EUP-e) comprise the lowest cultural layer (CL1) of the Tachikiri site (loc. Tachikiri, loc. Otsubobata, and loc. Imahira-Shimizu) and the Yokomine C site, all recovered just below Tane-IV and dated between 35,500–34,400 cal BP. The latter EUP sites (EUP-l) include the middle cultural layers (CL2) of the Tachikiri site (loc. Otsubobata, loc. Kozono, and loc. Imahira-Shimizu) and the Yokomine C site, dated between 34,400–30,000 cal BP. This latter phase lasted for more than 4,000 years, longer than the early phase (EUP-e). After the absence of archaeological data during the LGM, terminal LUP sites recovered above AT and below ICP-J components are include the Zenigame site, Naganosaki site, and upper cultural layers (CL3) of the Tachikiri site (loc. Tachikiri and loc. Otsubobata), all characterized by the microblade industry. The microblade industry in southern Kyushu Proper is dated between 17,800–16,100 cal BP as stated earlier, whereas there is no well-dated LUP component within Tanegashima. The Backed Point industry that prospered during the LGM in Kyushu Proper has not been found on Tanegashima Island so far. ICP-J sites, also recovered above AT and directly below Sz-S tephra, include the Onigano site, Okunonita site, and Sankakuyama I site. The Bayesian age model, established using a total of 25 radiocarbon dates of ICP-J sites (Table 2), indicates its time range between 14,340–12,730 cal BP at the maximum. Another point is that the ICP-J started around 14,340–14,073 cal BP, slightly older than has been previously presumed [37]. However, several dates, including the two oldest dates, have δ^13^C value slightly heavier than -24.0‰ (Table 2), indicating a potential marine reservoir effect [141, 142]. When excluding these dates with heavier δ^13^C value, the model shows the start of ICP-J at 14,243–13,849 cal BP, slightly younger than the above range.

Considering the synchronicity with the timing of the Greenland climatic fluctuation, the time range of EUP-e nearly corresponds with the GI-7 warming (35.48–34.74 ka BP) [143], and the onset of EUP-l with GS-7 stadial (34.74–33.74 ka BP) [143]. The onset of the ICP-J is several hundred years later than the onset of the GI-1 warming (14.69–12.89 ka BP) [143].

### Toolkit diversity, reduction technology, and lithic raw material

**Fig 8:**
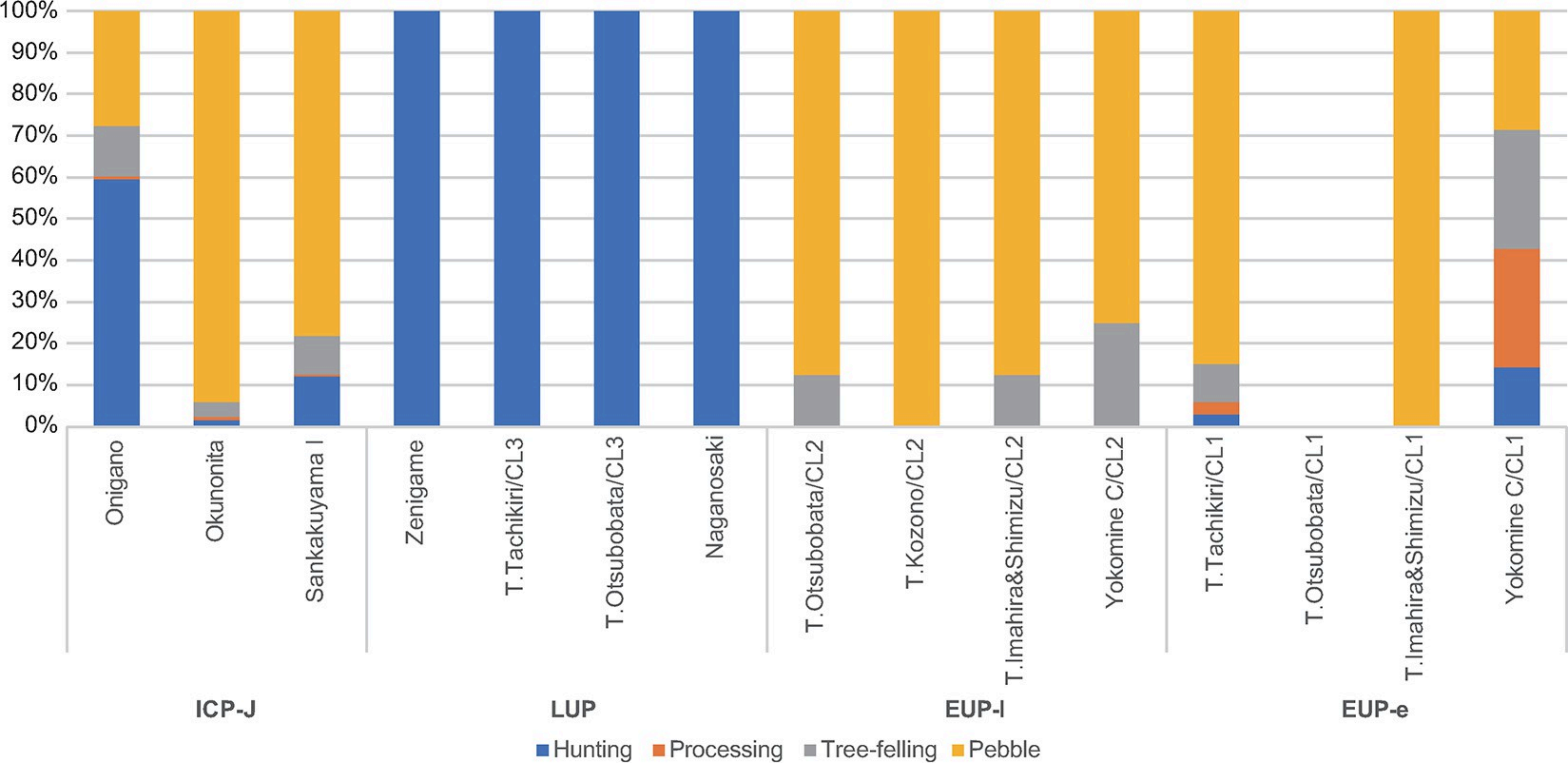
Tool category composition of each component.

Tool assemblage and toolkit diversity are detailed in Tables 3 and 4, while the composition of reduction technologies and lithic raw materials is summarized in Table 5. Tool categorical composition is summarized in Fig 8.

**Table 3:** Toolkit diversity of each component from EUP-e to EUP-l.

Tool Category	Tool Type	EUP-e components								EUP-l components							
		T. loc.Tachi-kiri/CL1		T. loc.Otsu-bobata/CL1		T. loc.Imahi-ra&Shimizu/ CL1		Yokomine C/CL1		T. loc.Otsu-bobata/CL2		T. loc.Kozo-no/CL2		T. loc.Imahi-ra&Shimizu/ CL2		Yokomine-C/ CL2	
		n	(%)	n	(%)	n	(%)	n	(%)	n	(%)	n	(%)	n	(%)	n	(%)
Hunting	Microblade																
	Trapezoid	2	3.0					1	14.3								
	Chipped arrowhead																
	Ground arrowhead																
	Bifacial/Unifacial point																
Processing	Denticulate	1	1.5					1	14.3								
	Scraper/Knife	1	1.5					1	14.3								
Tree-felling	Chipped axe	2	3.0														
	Ground axe	3	4.5														
	Chopper	1	1.5					2	28.6	2	12.5			2	12.5		
	Chopping tool															2	25.0
Pebble furniture	Stone plate	4	6.1							8	50.0	3	33.3	2	12.5		
	Stone plate+Anvil stone																
	Grinding stone	12	18.2			3	42.9					3	33.3	5	31.3	1	12.5
	Grinding+Hammer stone	25	37.9					1	14.3					1	6.3		
	Anvil stone	1	1.5									1	11.1			1	12.5
	Anvil/Grinding stone																
	Stone with surface pits	1	1.5														
	Whetstone	4	6.1			3	42.9			3	18.8	1	11.1				
	Whetstone/Hammer stone																
	Hammer stone	9	13.6			1	14.3	1	14.3	3	18.8	1	11.1	6	37.5	4	50.0
Total amount of classifiable tools		66		0		7		7		16		9		16		8	
Richness /	Evenness (SDI)	13	0.79	0	n/a	3	0.61	6	0.87	4	0.66	5	0.74	5	0.72	4	0.66
REV		10.3		n/a		1.8		5.2		2.6		3.7		3.6		2.6	
Percentage of recycled tool*		37.9		n/a		0.0		14.3		0.0		0.0		6.3		0.0	

*“Tachikiri site” is abbreviated as T.

*The recycled tool refers to tools showing multiple traces of use such as stone plate/anvil, grinding stone/hammer stone, anvil/grinding stone, and whetstone/hammer stone.

**Table 4:** Toolkit diversity of each component from TUP to ICP-J.

Tool Category	Tool Type	ICP-J components						LUP components							
		Onigano		Okunonita		Sankaku-yama I		Zenigame		T, loc.Tachikiri /CL3		T, loc.Otsubo-bata/CL3		Naganosaki	
		n	(%)	n	(%)	n	(%)	n	(%)	n	(%)	n	(%)	n	(%)
Hunting	Microblade							6	100.0	4	100.0			30	100.0
	Trapezoid														
	Chipped arrowhead	370	57.3	3	1.1	42	9.3					2	100.0		
	Ground arrowhead-	5	0.8	2	0.7	7	1.6								
	Bifacial/Unifacial point	10	1.5												
Processing	Denticulate														
	Scraper/Knife	4	0.6	2	0.7	2	0.4								
Tree-felling	Chipped axe	21	3.3	3	1.1	17	3.8								
	Ground axe	33	5.1	7	2.5										
	Chopper	21	3.3			20	4.4								
	Chopping tool	3	0.5												
Pebble furniture	Stone plate	55	8.5	16	5.7	69	15.3								
	Stone plate+Anvil stone	4	0.6												
	Grinding stone	43	6.7	165	58.5	11	2.4								
	Grinding+Hammer stone	32	5.0	3	1.1	129	28.7								
	Anvil stone	14	2.2			7	1.6								
	Anvil/Grinding stone	1	0.2	2	0.7										
	Stone with surface pits			5	1.8	1	0.2								
	Whetstone	7	1.1	4	1.4	67	14.9								
	Whetstone/Hammer stone	1	0.2												
	Hammer stone	22	3.4	70	24.8	29	6.4								
Total amount of classifiable tools		646		282		401		6		4		2		30	
Richness /	Evenness (SDI)	17	0.65	11	0.59	12	0.82	1	0.00	1	0.00	1	0.00	1	0.00
REV		11.1		6.5		9.8		0.0		0.0		0.0		0.0	
Percentage of recycled tool		5.9		1.1		32.2		0.0		0.0		0.0		0.0	

*“Tachikiri site” is abbreviated as T.

*The recycled tool refers to tools showing multiple traces of use such as stone plate/anvil, grinding stone/hammer stone, anvil/grinding stone, and whetstone/hammer stone.

**Table 5:** Frequency of five reduction technologies and presence of exotic lithic raw material in each component.

Phase	Site	Reduction technology					Exotic lithic raw material
		Flake	Pebble	Ground	Biface	Micro-blade	
ICP-J	Onigano	**54.7**	29.7	5.5	10.1	none	**present**
	Okunonita	1.7	**91.0**	3.1	4.1	none	**present**
	Sankakuyama I	12.0	**78.3**	5.6	4.0	none	**present**
LUP	Zenigame	none	none	none	none	**100.0**	absent
	Tachikiri; loc.Tachikiri/CL3	none	none	none	none	**100.0**	absent
	Tachikiri; loc.Otsubobata/CL3	**100.0**	none	none	none	none	absent
	Naganosaki	none	none	none	none	**100.0**	absent
EUP-l	Tachikiri; loc.Otsubobata/CL2	none	**100.0**	none	none	none	absent
	Tachikiri; loc.Kozono/CL2	none	**100.0**	none	none	none	absent
	Tachikiri; loc.Imahira &Shimizu/CL2	none	**100.0**	none	none	none	absent
	Yokomine C/CL2	none	**100.0**	none	none	none	absent
EUP-e	Tachikiri; loc.Tachikiri/CL1	5.8	**82.6**	4.3	7.2	none	absent
	Tachikiri; loc.Otsubobata/CL1	none	none	none	none	none	absent
	Tachikiri; loc.Imahira &Shimizu/CL1	none	**100.0**	none	none	none	absent
	Yokomine C/CL1	42.9	**57.1**	none	none	none	absent

*“Tachikiri site” is abbreviated as T.

*A tool type resulting from the combination of two reduction technologies for primary and secondary reductions is counted twice. For instance, ground axes are included in both the ’Biface’ and ‘Ground’ tool reduction categories.

The EUP-e components exhibit a small total number, with high variability among sites. The lithic toolkit predominantly includes low-mobility indicators such as pebble tools, alongside small numbers of flake tools like trapezoids and scrapers, all made of local lithic raw materials. The REV range from 10.3 to 1.8 across sites, with Tachikiri site loc.Tachikiri CL1 shows the highest REV.

EUP-l components are characterized by a low variety of lithic toolkits, mostly comprised of pebble tools. EUP-l components, however, exhibit lower inter-site variability compared to EUP-e with REV ranging narrowly between 3.7 and 2.6. Lithic tools during the EUP were typically produced through expedient flake reduction and pebble tool reduction using local sandstones. There is no evidence of the use of exotic raw materials.

The lowest value of toolkit diversity and the lowest inter-site variability were observed in terminal LUP components. Hunting weapons from all terminal LUP sites consisted solely of microblades made of locally available rounded cobble of shale, mudstone, and hornfels, indicating a highly standardized, maintainable tool procurement strategy for mobile lifeway, alongside the adoption of flake reduction technology (Table 5).

In contrast, ICP-J toolkits show a dramatic increase in diversity, with high REV ranging from 11.1 to 6.5. All sites exhibit a wide variety of tool types, including hunting weapons, processing tools, tree-felling tools, and cashed pebble tools in each site. ICP-J foragers adopted all reduction technologies except microblade technology. Pebble tool and flake reduction frequencies are notably high (Table 5). Each tool type is more elaborately manufactured compared to previous phases. Exclusively within ICP-J components, small flake tools (e.g., arrowheads) made from exotic lithic raw materials from Kyushu proper or Yakushima, such as obsidians and andesites, are identified (Table 5) [37, 38].

### Labor cost of constructing features

**Fig 9:**
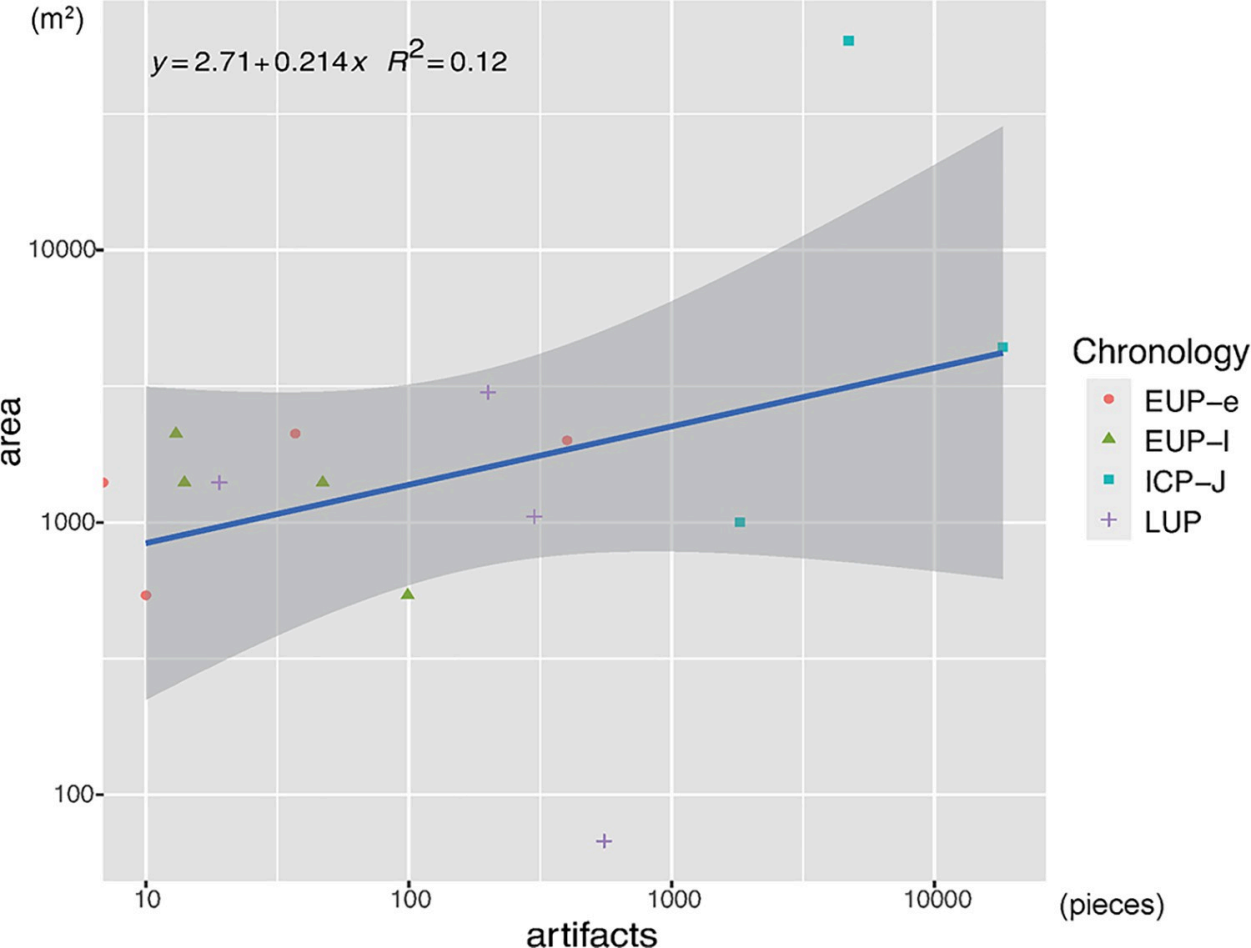
Number of recovered artifacts plotted against excavated area from the sites analyzed in this paper.

**Fig 10:**
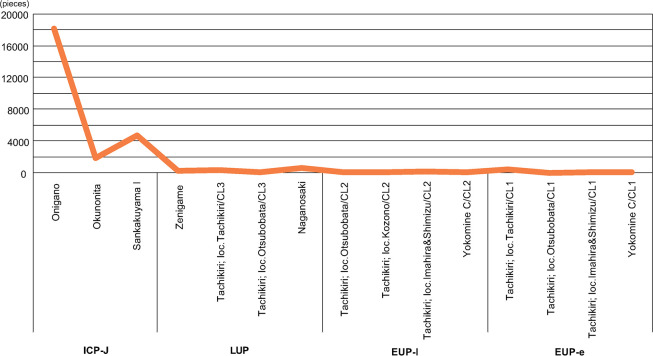
Diachronic change of total amount of artifact of each component and phase.

Fig 9 shows a weak correlation between excavated areas and assemblage sizes (R^2^ = 0.12), indicating that the consistently small number of artifacts throughout the Upper Paleolithic and the significant increase in artifact numbers in the ICP-J (Fig 10) are not strongly influenced by sampling bias.

The result of labor cost analysis for each site is summarized in Table 6. LCS values for EUP-e sites vary between 36 and 6. The highest LCS is recorded at the Tachikiri site. At Tachikiri site loc. Otsubobata and loc. Imahira-Shimizu, there are few lithic and pebble tools, but a total of 24 pit-traps are found. In contrast, Tachikiri site loc.Tachikiri CL1 exhibits a variety of features within the site area and contains a total of 76.23 kg of pebble tools used as site furniture or caches.

**Table 6:** Total weight of pebble tools, number of archaeological features, and LCS of each component.

Phase	Site	Total weight of pebble tools (kg)	Number of archaeological features*						LCS
			PH	PT	PA	SA	SP	BS/CC	
ICP-J	Onigano	386.77	5		4	5	6		44
	Okunonita	225.47			19	2	1		43
	Sankakuyama I	>82.00	2		4		1	1	17
LUP	Zenigame	0							0
	T, loc.Tachikiri/CL3	0							0
	T, loc.Otsubobata/CL3	0						3	0
	Naganosaki	0			1				2
EUP-l	T, loc.Otsubobata/CL2	43.47						13	0
	T, loc.Kozono/CL2	21.17			4			2	8
	T, loc.Imahira &Shimizu/CL2	13.85			1			7	2
	Yokomine C/CL2	4.30			6				12
EUP-e	T, loc.Tachikiri/CL1	76.23		2	1		13	31	21
	T, loc.Otsubobata/CL1	0		12					36
	T, loc.Imahira &Shimizu/CL1	4.54		12					36
	Yokomine C/CL1	0.34			3			10	6

*PH: pit-house, PT: pit-trap, PA: pebble aggregate, SA: stone alignment, SP: small pit, BS/CC: burnt soil or charcoal concentration

EUP-l witnessed a decline in LCS ranging between 12 and 2. Each site similarly includes pebble aggregates and/or burnt soils/charcoal concentrations, but no pit-traps were found during this period. Most artifacts recovered are pebble tools, with total weight ranging from 43.47 to 4.30 kg. Recycled or multi-functioned pebble tools, totaling 6.3%, were only observed at Tachikiri site loc. Imahira-Shimizu (Table 3).

Terminal LUP sites show a notable absence of pebble tools and features with high LCS values. Only a few burnt soils and pebble aggregates were recovered, with no evidence of tool recycling or heavily used tools.

**Fig 11:**
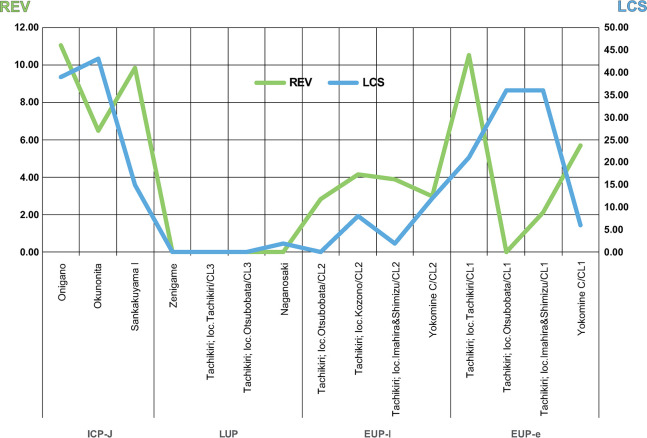
Diachronic change of REV and LCS values for each component and phase.

The ICP-J marked the highest LCS values, ranging from 44 to 17, with >82 kg~386.77 kg of pebble tools. Pit-houses were discovered at the Onigano and Sankakuyama I sites. The recycled or multi-functional tool ratio is higher than in the previous period. Of these, the Onigano site, the largest and best-preserved site, the LCS reached 44, followed by the Okunonita site at 43. The Onigano site yielded five pit-houses, four pebble aggregates, five stone alignments, a total of 3,822 lithic debitage, and 14,352 high-density potsherds from an excavated area of 4,400 m^2^. The lithic debitage included as many as 370 chipped arrowhead and more than 1,000 pieces of by-products, notably 265 bi-polar cores were reduced to obtain thin and small flakes to support of chipped arrowhead, using small, rounded shale and mudstone cobbles. Additionally, 386.77 kg of pebble tools and approximately 90 kg of unretouched pebbles, likely cached as blanks for hammer stones or grinding stones, were unearthed. In contrast, the Okunonita site had a higher number of pebble aggregates (n = 19) compared to the other sites, contributing to its high LCS score. The elevated LCS values also corresponds with large quantity of grinding stones (n = 165) and hammer stones (n = 70). The Diachronic changes in REV and LCS are summarized in Fig 11.

## Discussion

### Evaluating the mobility and sedentism

#### EUP-e

Although some EUP-e sites have high LCS values, EUP-e foragers allocated labor cost not to residential facilities but to pit-traps. The frequent formation of burnt soil and charcoal concentration does not suggest sedentism but rather a frequent reoccupation. The results indicate that the Tachikiri site was a repeatedly used camp with surrounding trap hunting locations. Yokomine C site CL1 also served these functions. Furthermore, these two components show a relatively high percentage of possibly recycled multi-functional tools (Tab.3), indicating intensified or recurring occupation. Because the vegetation during the EUP on Tanegashima was warm temperate forests during GI-7, it is suggested that foragers moved repetitively to specific sites, practicing sit-and-wait trap hunting with pit-traps and exploiting acorns from abundant forest resources [144]. This supposition aligns with starch residue analysis from the Tachikiri site of EUP-e, illustrating the utilization of plant resource on pebble tools [79]. All of these indicate that EUP-e hunter-gatherers, with reduced mobility targeting various resources, moved between settlements in the warm forested environment, possibly seasonally.

#### EUP-l

The subsistence strategy found in EUP-e, however, was discontinued in EUP-l, possibly due to the climatic cooling of GS-7 toward the LGM. Evidence from Tachikiri and Yokomine C site indicates that both REV and LCS gradually decreased during EUP-l. Foragers in EUP-l did not leave any evidence of pit-trap hunting. The percentage of recycled and multi-functional tools is generally low, illustrating a relative decrease of site occupational intensity and increased mobility toward LGM.

#### During the LGM

No archaeological sites assigned to the LGM have been found on Tanegashima. It is hypothesized that LGM foragers could not adapt to Tanegashima due to the limited availability of lithic raw materials required to make larger hunting weapons, based on archaeological data from contemporary Kyushu Proper. This is especially relevant if their subsistence relied on terrestrial animal hunting. Site-based paleoenvironmental data do not show a dense distribution of a warm forest rich in seeds during the LGM [75], which may have forced foragers to maximize their reliance on animal hunting and expand their foraging range [11, 12]. Additionally, the land area of Tanegashima (ca. 445 km^2^) may have been too small to support such highly mobile groups, resulting in the archaeological invisibility.

#### Terminal LUP

The terminal LUP foragers were equipped with mobile hunting toolkits featuring microblades. Unlike LGM foragers, the microblade technology allowed them to make necessary tools from small knappable rocks available on this island. Despite this innovation, the scarcity of lithic tools and low LCS strongly indicate highest mobility and the shortest patch exploitation time. Given their reliance on mobile hunting toolkit, terminal LUP foragers are inferred to be specialized mobile hunters, operating in both Tanegashima and Kyushu Proper across the land bridge, as suggested by the similarity in microblade technology. During this period, many small hunting camps without high LCS features are also found on Kyushu proper [44], implying foragers’ frequent residential movement.

#### ICP-J

The beginning of the ICP-J on Tanegashima is suggested to be around 14,243–13,849 cal BP, several hundreds of years later than the onset of the GI-1 warming and is rather closer to the estimated submersion timing of the Osumi Strait (e.g., submerged by 14,500 cal BP) in a context of flourishing evergreen forest. ICP-J foragers on Tanegashima had little commonality with LUP foragers in term of toolkit types and behaviors. They may have been new migrants across the sea from Kyushu Proper, pulled by the re-emergence of abundant resources on Tanegashima.

The drastic increase in the total amount of artifacts and the highest LCS indicate a quite sedentary nature of their settlement system, and the highest REV suggests they developed a wide variety of specialized toolkits, unsuitable for mobile lifeway. Their toolkits changed to include pebble tools for intensified utilization of plant food and bow and arrows for smaller mammals.

These results illustrate that foragers spent less time traveling between resource patches and adopted a broad diet including high-cost and low-return resource patches such as edible seeds. In this behavior, search time or procurement time was shorter and processing time became longer as described in the model as the ‘processor’, unlike the hunting-based society of ‘traveler’ in previous periods [110, 111].

The results from the Onigano site are noteworthy. Onigano, with the highest values of REV and LCS, indicates that the hunter-gatherers were highly sedentary and exploited a broad spectrum of resources from the warm temperate evergreen forest. This site provides the earliest evidence of such behavior in the late Pleistocene occupation history of Tanegashima. Onigano-type settlements are not found in central Tanegashima, possibly due to limited water availability. The Onigano site is located near the Kawawaki River, which has the largest drainage area on Tanegashima (Fig 3). The use of exotic lithic raw material at Sankakuyama I and especially at Onigano, suggests that these foragers maintained social relationship with people in Kyushu Proper. This aligns with the establishment of long-distance circulation of pottery and social networks, as illustrated by pottery analysis [37, 38]. Given that the inferred date of the onset of the ICP-J (14,243–13,849 cal BP) is close to the timing of the submergence of the Osumi Strait, we suggest that the foragers mitigated subsistence risks and the risk of isolation by engaging in high-cost long-distance exchange.

### Factors of decision making

Our analytical results provide a higher resolution understanding of the occupational history on Tanegashima than previous studies. Changing mobility patterns and resource procurement strategies are closely associated with resource variety and availability, in accordance with the fluctuation between warmer and cooler climatic conditions. This suggests that environmental factors may have primarily driven human behavioral decision-making.

This strong correlation between a mobility-sedentary continuum and the environmental context may suggest that sedentary lifeways could have been established during the Upper Paleolithic, although the evidence indicates otherwise. There is a significant difference in the total amount of artifacts and labor costs between the settlements of EUP-e and those of the ICP-J, with the latter showing a notably higher tendency (Figs 10 and 11). This demonstrates a remarkable increase in occupational intensity and signals the appearance of sedentary lifeway during the ICP-J. Moreover, the circulation of lithic raw materials and pottery between Tanegashima and Kyushu proper illustrates that ICP-J foragers established stable social interactions. These facts strongly indicate that the remarkable increase in occupational intensity needs to be explained not only by environmental factors but also by social aspects, such as increasing population size, possibly due to a population influx from Kyushu proper. This scenario is consistent with the anthropological implications that an increase in population size in a productive and stable environment is a key factor in the appearance of sedentism [145]. In this study, the ‘processor’ end of behavior co-occurs with high population density at the onset of ICP-J.

Lastly, focusing on the time and place of costly labor such as shelter construction, pebble aggregates, and pit-traps, these activities are concentrated at a limited number of sites. As partially evidenced by tool recycling and the repetitive use of features observed first in EUP-e and more prominently in ICP-J, the concentration of sites in limited areas, alongside the scarcity in other areas with similar resource availability, may limit the explanatory power of the traditional model and may suggest that the concept of a ‘constructed landscape’ is a better fit for the data [126]. This analysis also suggests that sites may not be as sedentary in areas with scarce water availability. Given the higher degrees of intra-site resource variability on Tanegashima than has been previously expected, more high-resolution research is necessary to better assess and refine the behavioral models.

## Conclusion

Abundant archaeological evidence from the southern island of the northwestern Pacific-rim, southern tip of the Paleo-Honshu Island, holds significant potential for unraveling the factors and processes of sedentism. The island of Tanegashima, with its unusually intact geochronology based upon tephrochronology, serves as a compelling example from the late Pleistocene temperate zone.

Our study illustrates that settlement-subsistence strategies on Tanegashima primarily correspond to the diachronic variability in environmental conditions, mainly food resources. During the EUP, a collector-like system with relatively high occupational intensity in productive forest ecosystems during the EUP-e shifted to a more mobile lifeways in the EUP-l in response to climatic cooling and possibly decreasing plant food resources. Eventually, foragers abandoned Tanegashima during the LGM due to the disappearance of productive forest, which forced them to rely more on animal hunting elsewhere. However, the island lacks lithic raw materials suitable for making large hunting weapons. The microblade technology of the terminal LUP provided a solution for this difficulty, allowing foragers to re-enter Tanegashima. The ICP-J sites reflect the increased resource availability in abundant forests, which relates to increased sedentism. This also suggests correlations, such as the case of the Onigano site, where a higher degree of sedentism was based on the more secure availability of fresh water.

Moreover, our study indicates that environmental conditions were not the sole determining factor of sedentism. Instead, the stability of sedentism strongly correlates with social factors, such as increasing population. The ICP-J sites on Tanegashima shows high occupation intensity and ‘processor’ behavior, suggesting a growing population, possibly due to an influx from Kyushu proper. This population growth, along with extended occupation duration, enabled ICP-J foragers to establish stable inter-group connections. Additionally, the occupation of specific locations may have led to constructed landscapes. Furthermore, increasingly sedentary in areas with predictable and abundant resources, along with enhanced social reproduction, likely engaged in long-distance social interaction to mitigate long-term resource and population risks [37, 38].

With this case study from Tanegashima Island, we agree with previous anthropological suggestions that the mobile-sedentary spectrum offers alternatives to manage risks of social and natural environments [1, 9, 11, 13, 145]. Various causative factors of sedentism include the expansion of diet breadth (Broad Spectrum Revolution) [15], resource intensification by changing projectile technology [17, 48, 146], and plant domestication [147]. The sedentism of ICP-J on Tanegashima was evidently accompanied by a complete technological change, expansion of diet breadth in the productive forest ecosystem, and increased population density. Although the relationship between cause and effect of these factors remains to be clarified in future work, our study provides insights into the fundamental causes of sedentism in the temperate forest during the late Pleistocene.

## Supporting information

S1 File S1. Current status of terminal Pleistocene archaeology in the Japanese Archipelago. S2. Geological setting and late Pleistocene tephrochronology of Tanegashima Island.
(DOCX)
