# Correction: More on mobility and sedentism: Changes in adaptation from Upper Paleolithic to Incipient Jomon, Tanegashima Island, southern Japan

**DOI:** 10.1371/journal.pone.0321869

**Published:** 2025-04-02

**Authors:** Kazuki Morisaki, Fumie Iizuka, Masami Izuho, Mark Aldenderfer

There are errors in the author contributions. The correct contributions are:

**Conceptualization:** Kazuki Morisaki, Fumie Iizuka, Masami Izuho, Mark Aldenderfer.

**Data curation:** Kazuki Morisaki, Fumie Iizuka, Masami Izuho.

**Formal analysis:** Kazuki Morisaki.

**Funding acquisition:** Kazuki Morisaki, Fumie Iizuka, Masami Izuho, Mark Aldenderfer.

**Investigation:** Kazuki Morisaki, Fumie Iizuka, Masami Izuho.

**Visualization:** Kazuki Morisaki.

**Writing – original draft:** Kazuki Morisaki, Fumie Iizuka, Masami Izuho, Mark Aldenderfer.
