# Supplementary material for: More on mobility and sedentism: Changes in adaptation from Upper Paleolithic to Incipient Jomon, Tanegashima Island, southern Japan
Source: PLoS One. 2025 Jan 27;20(1):e0314311. doi: 10.1371/journal.pone.0314311 (PMC11771872; doi:10.1371/journal.pone.0314311)
Supplement: S1 File — S2. Geological setting and late Pleistocene tephrochronology of Tanegashima Island. (DOCX) [file pone.0314311.s001.docx]

Supporting Information for

**More on Mobility and Sedentism: Changes in Adaptation from Upper Paleolithic to Incipient Jomon, Tanegashima Island, Southern Japan**

Kazuki Morisaki^1*^, Fumie Iizuka^2^, Masami Izuho^3^, Mark Aldenderfer^4^

^1^ Department of Archaeology, Graduate School of Humanities and Sociology, The University of Tokyo, Tokyo, Japan

^2^ Department of Anthropology, University of Wisconsin−Madison, Madison, Wisconsin, United States of America

^3^ Department of History and Archaeology, Faculty of Social Sciences and Humanities, Tokyo Metropolitan University, Tokyo, Japan

^4^ Department of Anthropology and Heritage Studies, University of California, Merced, California, United States of America

Supporting Information Text

**S1. Current status of terminal Pleistocene archaeology in the Japanese Archipelago**

The Japanese Archipelago is situated on the western Pacific Rim and spanning from subarctic to subtropical zones. Despite the dearth of favorable preservation conditions for organic materials such as bones, wood, fur, skin, and fibers in most regions of the Japanese archipelago, the past fifty years have witnessed an accumulation of an immense corpus of archaeological data from the late Pleistocene to the early Holocene sites (more than 12,500 sites) [1-3]. Given the enormous data, comprehending the foragers’ adaptation history in the archipelago could significantly contribute to a more profound understanding of the ongoing global discussion including the mobility and sedentism of Pleistocene foragers.

Recent reviews of terminal Pleistocene archaeology have characterized assemblages from the northern part of the archipelago (Paleo-Sakhalin-Hokkaido-Kuril Peninsula: Fig.1) as composed of highly mobile tools based on microblade, blade reduction, bifacial reduction along with flake reduction technologies [4-9]. On the other hand, those from the central part of the archipelago (Paleo-Honshu Island: Fig.1) are primarily based on a combination of flake and blade reduction technology [3,6,10-12]. Furthermore, Paleo-Ryukyu Islanders from the subtropical and tropical regions primarily utilized non-lithic tools such as shell implements, exploiting a wide variety of inland and coastal food resources including aquatic animals [13,14], thereby extending the antiquity of the adaptative patterns associated with coastal ecosystems [15] further into the Last Glacial. The geographical gradient of technological attributes in part suggests the relationship between human behavioral diversity and environmental conditions in this long islands.

Furthermore, the archipelago has earned recognitions as the region where, having the onset with the earliest adoption of pottery in the terminal Pleistocene, Jomon foragers established a long-standing sedentary lifestyle [16-18]. In contrast to the progress of AMS radiocarbon chronology on the earliest pottery and chemical analyses on the motivation of pottery adoption in East Asia, however, the insufficient effort of in-depth intra-regional studies on lithic technological variability, change in mobility-sedentary continuum impede the adequate assessment of both diachronic and synchronic behavioral diversity and underlying causes of diversity in the onset of sedentism. This is also in part due to the lack of theoretical discussion. This paper attempts to complement the lack of discussion by muti-faceted analyses on the degree of sedentism.

**S2. Geological setting and late Pleistocene tephrochronology of Tane Island.**

The basal geology of Tanegashima Island is composed of an accretionary complex with Eocene and Oligocene mudstones and sandstones, named Kumage group (Fig.3). Layers of Kumage group among upper Shimanto supergroup are, from the lower to the upper: the middle to late Eocene, turbidite composed of mudstone and tuff; the early to middle Eocene, sandstone and turbidites, and non-local pillow basalts; the late early Oligocene, turbidites predominated by sandstone with well sorted medium sized grains.

The Kukinaga formation is found mainly in the southeast of the island (Fig.3). From lower to the upper, there are conglomerates, mudstone, and sandstone dated to the mid-Miocene [19,20]. The Masuda formation in the central area dates to the Pliocene and is composed predominantly of sandstone (Fig.3)[20]. Lamprophyre, about 10 m in width and dated to 18.2 ± 0.9 Ma, intrudes in northern Tanegashima and extends for about 20 km from northeastern to southwestern Tanegashima [20]. Shimamazaki in southwestern Tanegashima has the occurrence of quartz porphyry and dates to the Miocene, 15.6 ± 0.8 Ma.

Tanegashima Island has no plutonic, obsidian, or andesite outcrops, and their absence facilitate distinction of locally produced and exotic artifacts recovered from the island context. From the existing bedrocks, small pebbles of hard shale, mudstone, and hornfels (φ<10 cm) is the only high-quality rocks suitable for manufacturing chipped stone tools.

Late Pleistocene tephra reported from Tanegashima Island are Ata, K-Tz, AT, Sz-S, and four tephra layers of Tane-I to IV (65,000 years ago to 35,000 yrs ago). Ata tephra (Ata), originating in what is now the southern Kagoshima Bay, is dated between 110,000-10,500 yrs ago, and is widely distributed throughout the southwestern Japan including Kyushu Proper, and also found on Yakushima and Tanegashima to the south [21]. The Kikai-Tozurahara tephra (K-Tz) from the Kikai Caldera, is dated to about 95,000 yrs ago. It is also found throughout southwestern Japan [21,22]. The Aira Tn Tephra (AT), from the Aira Caldera located in the northern Kagoshima Bay, is dated to about 30,000 cal yrs BP [21-24]. The wide distribution of AT is found throughout the Japanese Archipelago, the Sea of Japan, the southern Korean Peninsula, the East China Sea, and the Pacific Ocean Basin [21]. Sz-S from Sakurajima Volcano on the somma of Aira Caldera is dated to 12,800 cal yrs BP [21-23]. It is the largest among the tephra from Sakurajima Volcano located on the center of the present Kagoshima Bay. Tane-I and Tane-II, Tane-III, and Tane-IV are estimated to be 65 ka, 45 ka, 35 ka respectively, although the vents are not clarified yet [25].

**Supplementary Information References**

1. Japanese Palaeolithic Research Association. Palaeolithic Sites in the Japanese Islands: A Database. Tokyo: Japanese Palaeolithic Research Association; 2010.
2. Sato H, Izuho M, Morisaki K, Human cultures and environmental changes in the Pleistocene–Holocene transition in the Japanese Archipelago. Quat Int. 2011; 237(1/2):93-102. <https://doi.org/10.1016/j.quaint.2011.01.006>
3. Morisaki K. The evolution of lithic technology and human behavior from MIS 3 to MIS 2 in the Japanese Upper Paleolithic. Quat Int. 2012; 248:56-69. <https://doi.org/10.1016/j.quaint.2010.11.011>.
4. Nakazawa Y, Izuho M, Takakura J, Yamada S. Toward an Understanding of technological variability in microblade assemblages in Hokkaido, Japan. Asian Perspectives. 2005; 44(2):276-292. <http://doi.org/10.1353/asi.2005.0027>
5. Izuho M. Human Technological and Behavioral Adaptation to Landscape Changes around the Last Glacial Maximum in Japan: A Focus on Hokkaido. In: Graf KE, Ketron CV, Waters MR, editors. Paleoamerican odyssey. College Station: Texas A&M University Press; 2013. pp.45-64.
6. Morisaki K, Izuho M, Terry K, Sato H. Lithics and climate: technological responses to landscape change in Upper Palaeolithic northern Japan. Antiquity. 2015; 89(345): 554-572. <https://doi.org/10.15184/aqy.2015.23>
7. Nakazawa Y, Akai F, Late-Glacial bifacial microblade core technologies in Hokkaido: An implication of human adaptation along the northern Pacific Rim, Quat Int. 2016; 442B:43-54. <https://doi.org/10.1016/j.quaint.2016.07.019>
8. Fukuda M, Morisaki K, Sato H. Synthetic Perspective on Prehistoric Hunter-Gatherer. Adaptations and Landscape Change in Northern Japan. In: Cassidy J, Ponkratova I, Fitzhugh B, editors. Maritime prehistory of Northeast Asia. vol. 6. The archaeology of Asia-Pacific navigation. Singapore: Springer Verlag; 2022. pp.73-95. <https://doi.org/10.1007/978-981-19-1118-7_4>
9. Buvit I, Terry K, Izuho M. Pathways along the Pacific: Using early stone tools to. Reconstruct coastal migration between Japan and the Americas. In: Carson MT, editor. Paleolandscapes and Archaeology. London: Routledge; 2021. pp.39-81.
10. Morisaki K, Sano K, Izuho, M. Early Upper Paleolithic blade technology in the Japanese Archipelago. Archaeological Research in Asia. 2019; 17:79-97. <https://doi.org/10.1016/j.ara.2018.03.001>
11. Morisaki K. What motivated early pottery adoption in the Japanese Archipelago: A critical review. Quat Int. 2021; 608/609:65-74. <https://doi.org/10.1016/j.quaint.2020.10.006>
12. Izuho M, Kaifu Y. The appearance and characteristics of the Early Upper Palaeolithic in the Japanese Archipelago. In: Kaifu Y, Izuho M, Goebel T, Sato H, editors. Emergence and Diversity of Modern Human Behavior in Palaeolithic Asia. College Station: Texas A&M University Pres; 2015. pp.289-313.
13. Yamasaki S, Fujita M, Katagiri C, Kurozumi T, Kaifu Y. [Human use of marine shells from Late Pleistocene layers of Sakitari-do cave site, Nanjo city, Okinawa prefecture]. Anthropological Science. 2014; 122:9-27.
14. Fujita M, Yamasaki S, Sawaura R. The Migration, Culture, and Lifestyle of the Paleolithic Ryukyu Islanders. Pleistocene Archaeology – Migration, Technology, and Adaptation. IntechOpen. 2020 Dec; 1-15. <https://doi.org/10.5772/intechopen.92391>
15. Rick TC, Erlandson JM. Coastal Exploitation. Science. 2009; 325:952-953. <https://doi.org/10.1126/science.1178539>
16. Kobayashi T. Jomon reflections: Forager life and culture in the Prehistoric Japanese Archipelago. Oxford: Oxbow Books; 2003.
17. Pearson R. Jomon hot spot: increasing sedentism in south-western Japan in the Incipient Jomon (14,000–9250 cal. bc) and Earliest Jomon (9250–5300 cal. bc) periods. World Archaeol. 2006; 38(2):239-258. <https://doi.org/10.1080/00438240600693976>
18. Morisaki K, Oda N, Kunikita D, Sasaki Y, Kuronuma Y, Iwase A, Yamazaki T, Ichida N, Sato H. Sedentism, pottery and inland fishing in Late Glacial Japan: a reassessment of the Maedakochi site. Antiquity. 2019; 93:1442-1459. <https://doi.org/10.15184/aqy.2019.170>
19. Inoue K. The Neogene stratigraphy and microfossil dates of Kagoshima prefecture. In: The geological society of Japan, editor. The Abstract of the 96^th^ Annual Meeting of the Geological Society of Japan. Tokyo: The Geological Society of Japan, Tokyo; 1989. pp.171.
20. Ogasawara M. K-Ar age and geochemical characteristic of the Quartz-porphyry at Shimama, southern Tanegashima, and K-Ar age of a Lamprophyre from northern Tanegashima: implications for Miocene Igneous activities in the outer zone of Southwest Japan. J. Miner Petrol Sci. 1997; 92:454-464. <https://doi.org/10.2465/ganko.92.454>
21. Machida H, Arai F. Atlas of tephra in and around Japan. Tokyo: University of Tokyo Press; 2003.
22. Okuno M. Chronology of tephra layers in southern Kyushu, SW Japan, for the Last 30,000 years. Quaternary Research. 2002; 41(4):225-236.
23. Okuno M. Chronological study on widespread tephra and volcanic stratigraphy of the past 10,000 Years. Journal of Geological Society of Japan. 2019; 125(1):41-53. <https://doi.org/10.5575/geosoc.2018.0069>
24. Smith V, Staff R, Blockley S, Bronk Ramsey C, Nakagawa T, Darren M, Takemura K, et al. Identification and correlation of visible tephras in the Lake Suigetsu SG06 sedimentary archive, Japan: chronostratigraphic markers for synchronising of East Asian/West Pacific paleoclimatic records across the last 150 ka. Quat Sci Rev. 2013; 67:121-137. <https://doi.org/10.1016/j.quascirev.2013.01.026>
25. Okuno M, Kobayashi T. Late Pleistocene tephra layers distributed on Tane Island, southwest Japan. Quaternary Research. 1984: 33(2):113-117. <https://doi.org/10.4116/jaqua.33.113>
